# L1 Cell Adhesion Molecule-Specific Chimeric Antigen Receptor-Redirected Human T Cells Exhibit Specific and Efficient Antitumor Activity against Human Ovarian Cancer in Mice

**DOI:** 10.1371/journal.pone.0146885

**Published:** 2016-01-13

**Authors:** Hao Hong, Christine E. Brown, Julie R. Ostberg, Saul J. Priceman, Wen-Chung Chang, Lihong Weng, Paul Lin, Mark T. Wakabayashi, Michael C. Jensen, Stephen J. Forman

**Affiliations:** 1 Department of Cancer Immunotherapeutics & Tumor Immunology and Hematology & Hematopoietic Cell Transplantation, Beckman Research Institute, City of Hope National Medical Center, Duarte, California, United States of America; 2 Department of Gynecologic Oncology, Beckman Research Institute, City of Hope National Medical Center, Duarte, California, United States of America; 3 Ben Towne Center for Childhood Cancer Research, Seattle Children’s Research Institute, Seattle, Washington, United States of America; University of Pittsburgh, UNITED STATES

## Abstract

New therapeutic modalities are needed for ovarian cancer, the most lethal gynecologic malignancy. Recent clinical trials have demonstrated the impressive therapeutic potential of adoptive therapy using chimeric antigen receptor (CAR)-redirected T cells to target hematological cancers, and emerging studies suggest a similar impact may be achieved for solid cancers. We sought determine whether genetically-modified T cells targeting the CE7-epitope of L1-CAM, a cell adhesion molecule aberrantly expressed in several cancers, have promise as an immunotherapy for ovarian cancer, first demonstrating that L1-CAM was highly over-expressed on a panel of ovarian cancer cell lines, primary ovarian tumor tissue specimens, and ascites-derived primary cancer cells. Human central memory derived T cells (T_CM_) were then genetically modified to express an anti-L1-CAM CAR (CE7R), which directed effector function upon tumor antigen stimulation as assessed by *in vitro* cytokine secretion and cytotoxicity assays. We also found that CE7R^+^ T cells were able to target primary ovarian cancer cells. Intraperitoneal (i.p.) administration of CE7R^+^ T_CM_ induced a significant regression of i.p. established SK-OV-3 xenograft tumors in mice, inhibited ascites formation, and conferred a significant survival advantage compared with control-treated animals. Taken together, these studies indicate that adoptive transfer of L1-CAM-specific CE7R^+^ T cells may offer a novel and effective immunotherapy strategy for advanced ovarian cancer.

## Introduction

Ovarian cancer is the most lethal among all gynecological malignancies, and is responsible for the majority of gynecologic cancer deaths, with an estimated 14,030 deaths in 2013 [1]. Despite improvements in surgical approaches and the refinements of frontline cytotoxic combinations over the past two decades, the majority of patients in advanced stages of disease at the time of diagnosis eventually succumb to tumor recurrence [2]. Thus, novel therapeutic approaches are desperately needed. With the growing recognition that ovarian tumors are immunogenic, and can be recognized and attacked by the immune system, various immune-based modalities have been actively explored to augment the efficacy of conventional therapies with the potential to prevent recurrence. Indeed, a number of peptide vaccines, dendritic cell vaccines and adoptive cell therapy strategies have been examined in clinical trials (reviewed in [3]).

The recent clinical efficacy of chimeric antigen receptor (CAR)-based adoptive T cell immunotherapy in the treatment of subsets of patients with acute lymphoblastic leukemia, and chronic lymphocytic leukemia (reviewed in [4, 5]) has provided important support for extending this form of immunotherapy to the treatment a wider scope of malignancies. CARs are unique in endowing T cells with cytotoxic effector functions in an HLA-unrestrictive manner, and thus are not subject to tumor escape as a consequence of HLA downregulation (reviewed in [6]). This is particularly important in ovarian cancer, where advanced disease is correlated with HLA downregulation [7]. Indeed, efforts to design CAR T cells for the treatment of ovarian cancer has been the focus of several preclinical and clinical studies. Preclinical anti-tumor activity against ovarian tumors has been reported using T cells expressing CARs specific for mesothelin [8] and MUC16 [9]. Folate receptor-specific CAR-modified T cells have been tested in a phase I trial for recurrent ovarian cancer, but lack of T cell persistence and localization to the tumor, as well as lack of tumor regression suggests that the strategy requires further optimization [10].

We and others have shown that the L1-cell adhesion molecule (L1-CAM) is highly over-expressed in ovarian cancer, while absent in normal ovaries [11, 12], and that its expression on tumors is associated with poor clinical outcome [13–15]. Previous studies have also reported that monoclonal antibodies directed against L1-CAM inhibit the growth of solid tumor cells *in vitro* and the growth of SKOV3 human ovarian carcinoma cells in a human xenograft model [16]. These data, along with our previous experience using cytotoxic T lymphocytes expressing a CAR specific for the CE7 epitope of L1-CAM (CE7R) to treat children with advanced refractory neuroblastoma [17], has resulted in our interest in examining the utility of CE7R^+^ T cells as a potential immunotherapeutic strategy in ovarian cancer.

## Materials and Methods

### Tumor cell lines

Ovarian adenocarcinoma lines CAOV-3, OVCAR-3, SK-OV-3, MADH2780, and A2780 were obtained from the American Type Culture Collection (ATCC) and cultured under ATCC suggested conditions. Generation of the EBV-transformed lymphoblastoid cell line that expresses a membrane tethered OKT3 single chain antibody (LCL-OKT3) was previously described [18]. Firefly luciferase-positive SK-OV-3 cells (ffluc+ SK-OV-3) were generated by lentiviral transduction of SK-OV-3 cells with an eGFP-ffLuc_epHIV7 lentiviral vector at a multiplicity of infection (MOI) of 5. Cryopreserved banks of all cell lines were authenticated for the desired antigen/marker expression by flow cytometry, and thawed cells were cultured for less than 6 month prior to use in assays.

Freshly-isolated ascites fluid from ovarian cancer patients who underwent paracentesis was obtained from City of Hope National Medical Center (COHNMC) surgical staff in a sterile vacuum container with the approval of the COHNMC Institutional Review Board (IRB) and Office of Human Subjects Protection. The COHNMC IRB waived the need for written informed consent as all samples were de-identified and ascites was discard material. The ascites was then immediately placed in culture with an equal volume of MCDB105 (Sigma)/M199 (Gibco) medium supplemented with 10% fetal bovine serum (HyClone), and penicillin/streptomycin (Fisher Scientific). Ovarian tumor cells eventually adhered to the cell culture plate surface. All experiments using primary cells were performed within two culture passages.

### DNA constructs and lentiviral vector

The CE7R28Z-T2A-CD19tDHFR^FS^_epHIV7 and CE7R(EQ)28Z-T2A-CD19t_epHIV7 lentiviral constructs used to generate CE7R^+^ cells contained a) the chimeric antigen receptor (CAR) sequence consisting of the V_H_ and V_L_ gene segments of the L1-CAM-specific CE7 mAb [19], an IgG4 hinge-CH2-CH3 sequence either with or without the L235E and N297Q double mutation (EQ) to prevent FcR binding and thus improve persistence in NSG mice [20], the transmembrane and cytoplasmic signaling domains of the costimulatory molecule CD28 that contains gg mutations to enhance CAR expression and function [21], and the cytoplasmic domain of the CD3ζ chain [22]; b) the ribosomal skip T2A sequence [23]; and c) the truncated human CD19 (CD19t) transduction selection marker sequence which lacks the cytoplasmic signaling tail (truncated at amino acid 323), in some cases directly followed by the double mutated human dihydrofolate reductase gene (L22F, F31S; DHFR^FS^), which confers resistance to immunosuppressive drugs of methotrexate (MTX) [24]. The GM-CSF receptor alpha chain signal sequence was used for both a) and c) to drive cell surface expression of the CAR and selection genes. The CD19R(EQ)28Z-T2A-EGFRt-T2A-DHFR^FS^_epHIV7 lentiviral construct used to generate CD19-specific CAR (CD19R+) T_CM_ contained the same indicated components as described above for the CE7R-containing constructs, with the exceptions of the CAR, which has the V_H_ and V_L_ gene segments of the CD19-specific FMC63 mAb [25], and the use of a truncated human EGFR (EGFRt) as transduction selection marker [26]. All construct and construction associated polymerase chain reaction primer sequences are available upon request.

### Activation, lentiviral transduction, and expansion of T_CM_

Human peripheral blood mononuclear cells (PBMCs) were isolated as described [18] from heparinized peripheral blood obtained from healthy donor leukapheresis products or discard kits containing residual blood components of healthy donors that had undergone apheresis at the COHNMC with the approval of the COHNMC IRB and Office of Human Subjects Protection. Blood samples from healthy donor leukapheresis products were obtained with written informed consent. The COHNMC IRB waived the need for written informed consent of blood samples from healthy donor leukapheresis discard kits as these were de-identified and obtained from discard material. T_CM_ isolation (using CD14- and CD45RA-depletion followed by CD62L-selection), anti-CD3/CD28 bead stimulation, and lentiviral-mediated transduction were then done as previously described [27] using MOIs ranging from 1 to 3. The Dynabeads Human T Expander CD3/CD28 (Invitrogen) were removed between days 10 and 14, and bead-free T cells were expanded at a cell density of 0.5–1 x10^6^ cells/mL with cytokines [27]. In some cases the cells transduced with CE7R28Z-T2A-CD19tDHFR^FS^_epHIV7 underwent further expansion (Rapid expansion method; REM) as previously described [18] with or without DHFR^FS^-mediated selection using 0.1 μM methotrexate (MTX) [24].

### Flow cytometry

Cell-surface L1-CAM was evaluated with purified CE7 mAb [12] followed by phycoerythrin (PE)-conjugated goat anti-mouse IgG (BD Biosciences). CAR (CE7R or CD19R) expression was evaluated with biotinylated Donkey anti-human Fcγ (Jackson ImmunoResearch Laboratories) followed by PE-conjugated streptavidin (SA-PE, BD Biosciences). Alternatively, transgene expression was evaluated by detecting the CD19t or EGFRt markers with either PE-conjugated anti-CD19 (Beckman Coulter) or biotinylated-cetuximab [26] followed by SA-PE, respectively. CD4 and CD8 expression was detected using fluorochrome-conjugated anti-CD4 and anti-CD8 (BD Biosciences). Flourochrome-conjugated isotype-matched mAbs served as controls (BD Biosciences). Data acquisition was performed on a FACScalibur (BD Biosciences) and percentages of immunoreactive cells were calculated using FCS Express version 3 software (De Novo Software).

### Immunohistochemical staining

Immunohistochemical evaluation of L1-CAM expression via CE7 mAb was performed as previously described [12].

### Immunofluorescence confocal microscopy

Freshly obtained ascites cells were directly deposited onto slides using a Shandon Cytospin 4 cytocentrifuge, fixed in 4% paraformaldehyde, permeabilized in 0.1% Triton X-100, and stored in PBS at 4°C until stained overnight with either CE7 mAb [12] or keratin 18 mAb (Cell Signaling) at 4°C, followed by Alexa Fluor 488–conjugated goat-anti-mouse IgG staining at room temperature for one hour. Nuclei were counter-stained with DAPI. Negative controls were stained in parallel by using secondary antibody only. Cells were examined on a Zeiss upright LSM 510 2-photon microscope. Images were acquired with a Zeiss plan-neofluar 20/0.5air lens or plan neofluar 40/1.3 numerical aperture oil immersion lens, and fields of view were then analyzed using the Zeiss LSM Image Browser.

### Western Analysis

Western blotting analysis for CE7R expression was performed using an anti-human CD3ζ chain (cytoplasmic tail)-specific monoclonal antibody 8D3 (BD Pharmingen), followed by goat anti-mouse IgM (mu chain) IRDye^®^ 800CW conjugated secondary antibody (Rockland Immunochemicals Inc), and imaging with the Odyssey Infrared Imaging System (LI-COR).

### Cytotoxicity and cytokine production assays

4-hour ^51^Cr release and inflammatory cytokine release assays were performed as previously described [12].

### Xenograft ovarian tumor model and xenogen imaging

Eight week-old female NOD/scid-IL2Rγnull (NSG) mice were inoculated i.p. with 3x10^4^ ffLuc^+^ SK-OV-3 cells at day 0. Tumor engraftment was evaluated by Xenogen non-invasive optical imaging as previously described [28], and mice with progressively growing tumors were segregated into different groups to receive two i.p. injections of either 5 x 10^6^ CE7R^+^ T_CM_ (14.7 x 10^6^ total cells), 14.7 x 10^6^ mock-transduced T cells, or PBS on days 5 and 12. Optical imaging was continued after adoptive T cell therapy to monitor tumor growth. Humane endpoints were used during the animal survival study, with mice euthanized by CO_2_ inhalation upon signs of distress such as distended belly due to tumor ascites formation, seizures, tremors, labored or difficulty breathing, weight loss (>20% body weight), signs of emaciation (i.e., prominent skeletal structures), impaired ambulation, inability to remain upright, or evidence of being moribund. Mice were monitored up to once a day as the survival study progressed, with no unexpected deaths. All mouse experiments were reviewed and approved by the COHNMC Institute Animal Care and Use Committee.

### Fluorescence in situ hybridization (FISH)

Cells were exposed to 0.075M KCl at 37°C for 20 minutes, fixed with Carnoy’s fixative, and dropped on slides. Slides were then incubated in 2XSSC at 37°C for 30 minutes, dehydrated through ethanol series, air dried and the following probes were applied: LSID13S319/LSI 13q34, CEP4, CEP10, and CEP 17 (Abbott Molecular), and homebrew probes for chromosome 5 and 9. BAC 53k22 (5p15) and P1-1069 (CDKN2A) were labeled in Spectrum Orange (Abbott Molecular). BAC 98O22 (5q31) and PACS 835j22 and 1132h12 (ABL) were labeled in Spectrum Green (Abbott Molecular). After overnight hybridization at 37°C, slides were washed in 0.4XSSC/0.1% Igepal at 72°C for 2 minutes, followed by 2XSSC/0.3% Igepal at room temperature for 2 minutes, counterstained with DAPI (Vector Laboratories) and imaged on the Bioview Duet Image Analyzer. A minimum of 200 cells was scored per sample.

### Statistical analysis

Unpaired Student's t-test was used to evaluate differences in flux values via Xenogen Living Image (photons/sec). Kaplan—Meier survival curves were compared using the log-rank test. GraphPad Prism 6.0 (GraphPad Software) was used for these statistical calculations. Values of P < 0.05 were considered significant.

## Results

### L1-CAM is a clinically relevant ovarian cancer associated antigen

**Fig 1:**
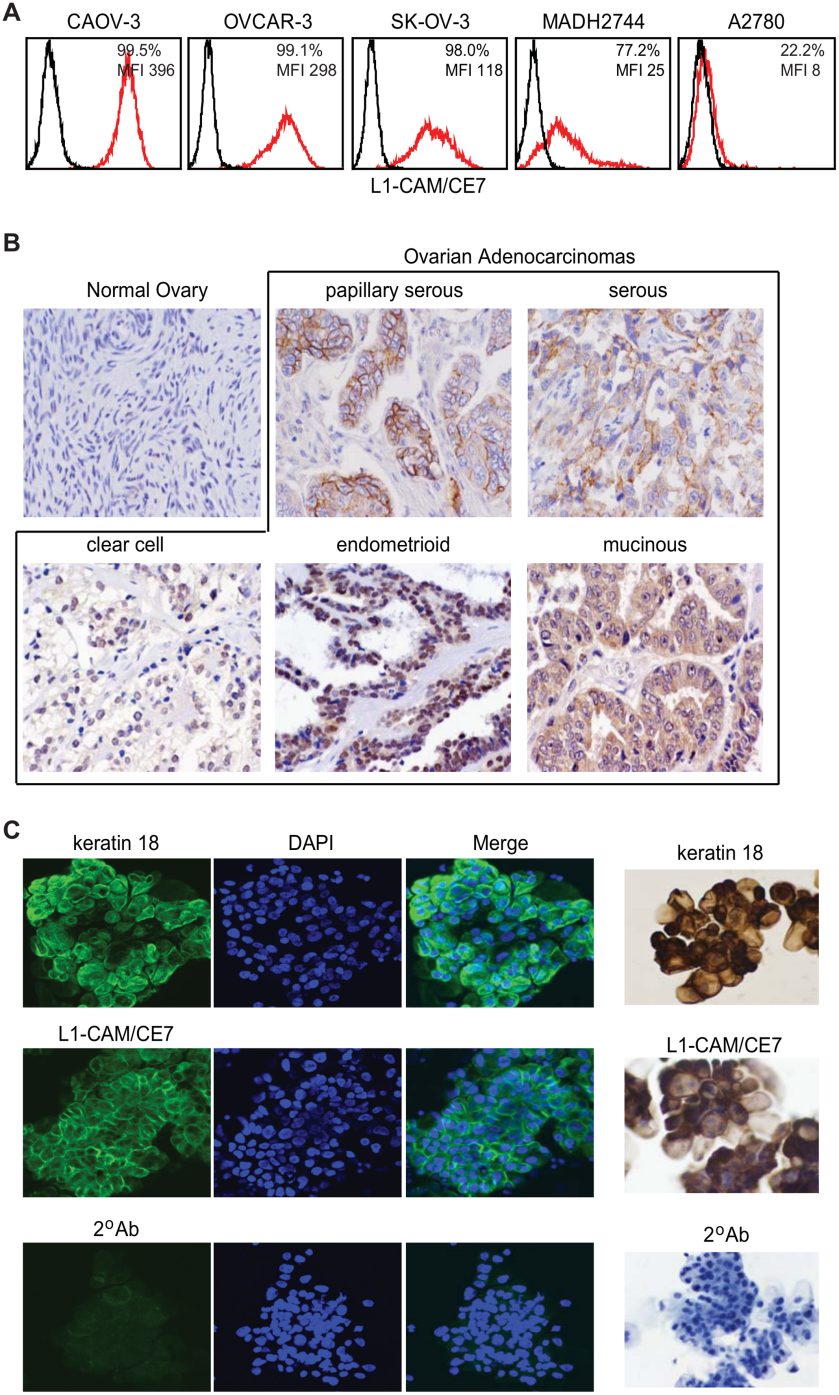
The CE7 Epitope of L1-CAM is a Clinically Relevant Ovarian Cancer Associated Antigen. **A**, Cell-surface expression of L1-CAM in various ovarian tumor lines including CAOV-3, OVCAR-3, SK-OV-3, MADH2744, and A2780 were examined by flow cytometry via CE7 mAb. Mean fluorescent intensity (MFI) and percentages of cells with positive staining (%, red histograms) over secondary reagent alone (black histograms) are indicated. **B**, Human ovarian cancer tissue microarray of primary and metastatic tumors was immunohistochemically stained with CE7 mAb. Representative images from each histological subtype are depicted. Photomicrographs are shown at 400x magnification (ocular 10x; objective 40x). **C**, Fresh ascites cells derived from ovarian cancer patients were immunofluorescently stained (*Left*) using anti-keratin 18 mouse or CE7 mAb followed by Alexa Fluor 488–conjugated goat-anti-mouse IgG. Nuclei were counter-stained with DAPI (blue). Ascites cells were also immunohistochemically stained (*Right*) with the same primary antibodies. In each case, staining with goat-anti-mouse secondary antibody alone (2° Ab) served as negative controls.

To further evaluate L1-CAM as a target antigen on ovarian cancer cells, we first screened cell-surface expression of the CE7 epitope of L1-CAM on five ovarian cancer cell lines by flow cytometric analysis. The CE7 epitope of L1-CAM has been suggested to have tumor-restricted expression, as it has been detected on various tumor tissues and malignant cell lines, while many normal tissues do not express this antigen, and some non-malignant cells, like monocytes, express L1-CAM, but not its CE7 epitope [12]. Out of the five ovarian cancer lines investigated here, four of them, OVCAR-3, SK-OV-3, MADH2744 and CAOV-3, expressed high levels of L1-CAM/CE7 whereas A2780 cells exhibited little to no L1-CAM/CE7 expression (Fig 1A), expanding on our previous findings [12].

L1-CAM/CE7 expression was next evaluated on different histological subtypes of primary patient-derived ovarian tumor specimens by immunohistochemical staining of 40 cases of ovarian tumor tissue microarray [12]. Positive immunoreactivity of L1-CAM/CE7 was detected in 39 cases [12], and spanned all four histological subtypes investigated including serous, clear cell, endometrioid, and mucinous (Fig 1B). There was considerable heterogeneity in L1-CAM/CE7 expression between tumor samples, including the proportion of tumor cells expressing L1-CAM/CE7, and the intensity of the L1-CAM staining. Of note, L1-CAM/CE7 was not detected on all samples of normal ovary, but found at varying extents on most subgroups of ovarian adenocarcinomas (Fig 1B).

L1-CAM/CE7 expression on fresh ovarian cancer cells directly derived from ascites fluid of patients who underwent therapeutic paracentesis was also examined. To distinguish from possible contaminating non-tumor cells that might also present in ascites fluid, such as stromal fibroblasts, red blood cells, leukocytes, etc, staining for the epithelia-specific cytokeratin (keratin 18) was also performed since ovarian cancer is generally thought to be epithelial in nature. Both immunofluorescent (Fig 1C **Left**) and immunohistochemical (Fig 1C **Right**) staining demonstrated that the cells from ascites fluid expressed keratin 18, which suggested they were exfoliated cancer cells, and many were also highly positive for L1-CAM/CE7. Further, L1-CAM expression was confirmed on positive and negative cells and tissues using the pan-specific L1-CAM antibody clone UJ127.11, and no significant differences in the expression between the CE7 epitope and total L1-CAM were observed, confirming the presence of the CE7 epitope for L1-CAM-expressing ovarian cancers (data not shown and [12]). Taken together, these findings along with others using L1-CAM detecting reagents that were not specific for the CE7 epitope [29, 30], validate the potential for L1-CAM, including its CE7 epitope, as an immunotherapeutic target for ovarian cancer.

### CE7R^+^ T_CM_ exhibit *in vitro* effector activity against L1-CAM^+^ ovarian cancer cell lines

**Fig 2:**
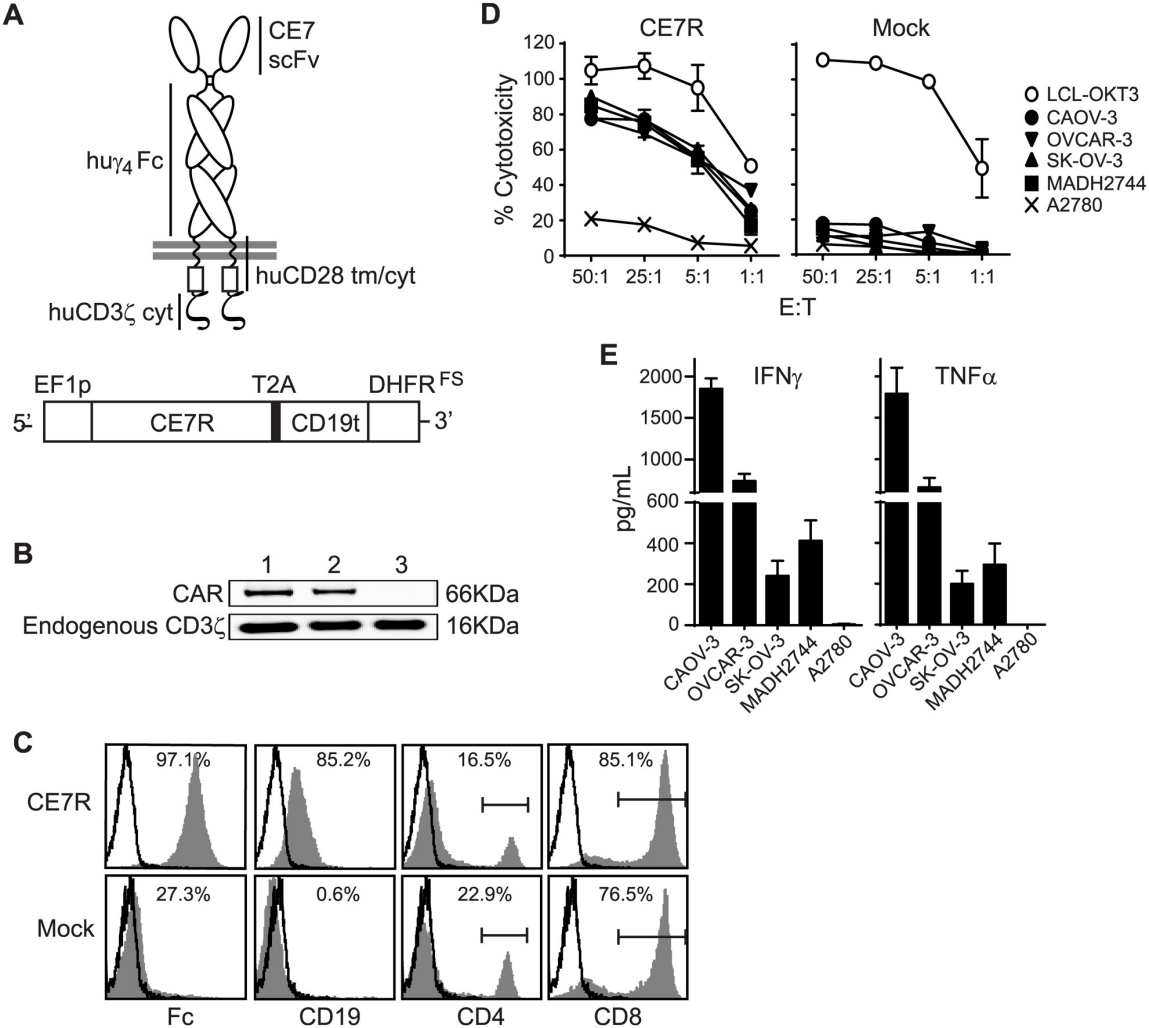
CE7R^+^ T_CM_ Cells Specifically Target L1-CAM Positive Tumor Cells *in Vitro*. **A**, Schematic representation of the second generation CE7-specific CAR (top) and a representative lentiviral CE7R cassette (bottom) which contains sequences coding an immunoglobulin single chain variable fragment (scFv) derived from the L1-CAM-specific murine CE7 monoclonal antibody linked through a human immunoglobulin (huγ_4_ Fc) hinge region to the human CD28 transmembrane and cytoplasmic signaling domains (huCD28 tm/cyt), and the human CD3 ζcytoplasmic (huCD3ζ cyt) domain followed by the ribosomal skip T2A sequence, and selection markers CD19t and double mutant DHFR (DHFR^FS^). Construct expression is driven by an EF-1α promoter. **B**, Western blots revealing both the endogenous CD3ζ (internal loading control) and the CE7R bands detected with anti-human CD3 ζ cytoplasmic tail-specific antibody. Lane 1: a known CAR-positive T cell line (positive control), Lane2: CE7R-transduced T_CM_ cells, Lane3: mock-transduced T_CM_ cells. **C**, Flow cytometric analysis of surface expressed Fc-containing CE7R, CD19t, CD4, or CD8 (grey histogram) compared to staining with either isotype controls or SA-PE alone (open histograms). **D**, Cytolytic activity of CE7R^+^ T_CM_ cells against the indicated ovarian cancer cell line targets was determined by 4-hr ^51^Cr-release assay. LCL-OKT3 was used as positive control target. Mean % chromium release ± S.D. of triplicate wells are depicted. **E**, CE7R+ T_CM_ cells were co-cultured with the indicated tumor lines at a 10:1 ratio for 21 hrs and supernatants were analyzed for IFN-γ and TNF-α levels by cytometric bead array. Means + S.E.M. of triplicate wells are depicted.

Building on our prior experience with first-generation CE7-specific CAR T cells that specifically target L1-CAM [12, 17, 19], we developed a second-generation CAR that contains the previously reported L1-CAM/CE7-binding scFv [19] in conjunction with a CD28 costimulatory domain in series with the signaling domain of CD3ζ to increase potency [22, 31, 32] (Fig 2A). The CAR lentiviral cassette also included a T2A ribosomal skip sequence [23] followed by a truncated CD19 selection/tracking marker (CD19t) to facilitate identification of successful CAR-expressing T cells (Fig 2A). In some cases, a mutant dihidrofolate reductase (DHFR^FS^) sequence was included in conjunction with the CD19t (Fig 2A) to confer methotrexate (MTX) resistance and thus allow for *in vitro* selection of transductants. For these studies, we utilized enriched central memory T cells (T_CM_) (CD45RA-, CD62L+) [27], as our group has developed a clinical manufacturing platform for this memory subset that has been previously shown to have favorable properties for therapeutic application, including long-term persistence upon adoptive transfer *in vivo* [18, 33, 34]. Primary human T_CM_ enriched from healthy donors that were lentivirally transduced to express the L1-CAM-specific CAR (CE7R) and expanded with one cycle of REM, were examined for CAR protein expression by Western blotting (Fig 2B). The endogenous CD3ζ chain of 16KDa was detected in both transduced and mock-transduced T cells, while the approximate 66 kDa band indicative of the CAR was only present in CE7R-transduced T cells (Fig 2B). Stable cell surface expression of the CE7R (as detected by anti-Fc antibody) and CD19t immunologic marker on transduced T_CM_-derived cells that had been expanded in the presence of MTX was confirmed via flow cytometric analysis (Fig 2C). Transduction and selection of CAR-expressing cells did not significantly alter the CD4/8 ratios as compared to mock-transduced controls (Fig 2C).

Next, the CE7R^+^ T cells were assessed for their effector function upon tumor antigenic stimulation. The CE7R^**+**^ T cells exhibited robust re-directed cytolytic activity against all tested L1-CAM^+^ ovarian cancer cell lines (i.e., CAOV-3, OVCAR-3, SK-OV-3, and MADH2744), but did not target the L1-CAM negative ovarian cancer cell line, A2780, in standard 4-hour ^51^Cr release assays (Fig 2D). Interestingly, this cytolytic activity did not appear to correlate with L1-CAM/CE7 expression level, as the ‘low expressor’ MADH2784 (Fig 1A) was killed with similar efficiency as the other three L1-CAM^+^ ovarian tumor lines. In contrast, mock-transduced parental T cells did not lyse any of the targets except the human lymphoblastoid cell line that expresses membrane bound CD3-agonistic antibody OKT3 (LCL-OKT3), which was used as a positive control target to depict the maximal cytolytic potential of the T cells. *In vitro* stimulation assays similarly revealed that the CE7R^+^ T cells produced significant amounts of pro-inflammatory cytokines such as IFN-γ and TNF-α upon co-culture with all L1-CAM^+^ ovarian tumor cell lines, but not upon stimulation with the L1-CAM-negative A2780 cells (Fig 2E). The cytokine response, in contrast to the killing assays, demonstrated some relationship to L1-CAM expression intensity, where CAOV-3 cells induced the highest and SK-OV-3 and MADH2744 induced the lowest cytokine levels. Together these data demonstrated that CE7R-expressing T cells can be triggered to exert L1-CAM-specific effector activity *in vitro* against target ovarian cancer lines.

### Adoptively transferred CE7R^+^ T_CM_ exhibit potent *in vivo* anti-tumor activity

To examine the anti-tumor activity of CE7R-expressing T_CM_ in preclinical mouse models, we developed a human xenograft model of intraperitoneal ovarian tumor using an SK-OV-3 cell line that was engineered to express firefly luciferase (ffLuc^+^ SK-OV-3) to allow for monitoring of tumor growth in mice with bioluminescence imaging. Given the fact that even advanced ovarian tumors remain largely confined within the peritoneal cavity and rarely disseminate via the vascular system in patients, we chose to administer tumor cells directly into the peritoneal cavity to model such clinically relevant intraperitoneal (i.p.)-disseminated ovarian cancer. Inoculation of NSG mice with ffLuc^+^ SK-OV-3 cells i.p. led to development of one large primary tumor nodule and dissemination of multiple smaller nodular tumors to the organs within in the peritoneal cavity, omentum and mesentery, and/or the animals presented with distended abdomens filled with bloody ascites (up to 3mL per mouse) within 1.5 to 2 months. Development of peritoneal carcinomatosis, dissemination of the tumor cells throughout the abdomen, and massive ascites in this xenograft model closely mimics clinical situations in ovarian cancer patients.

**Fig 3:**
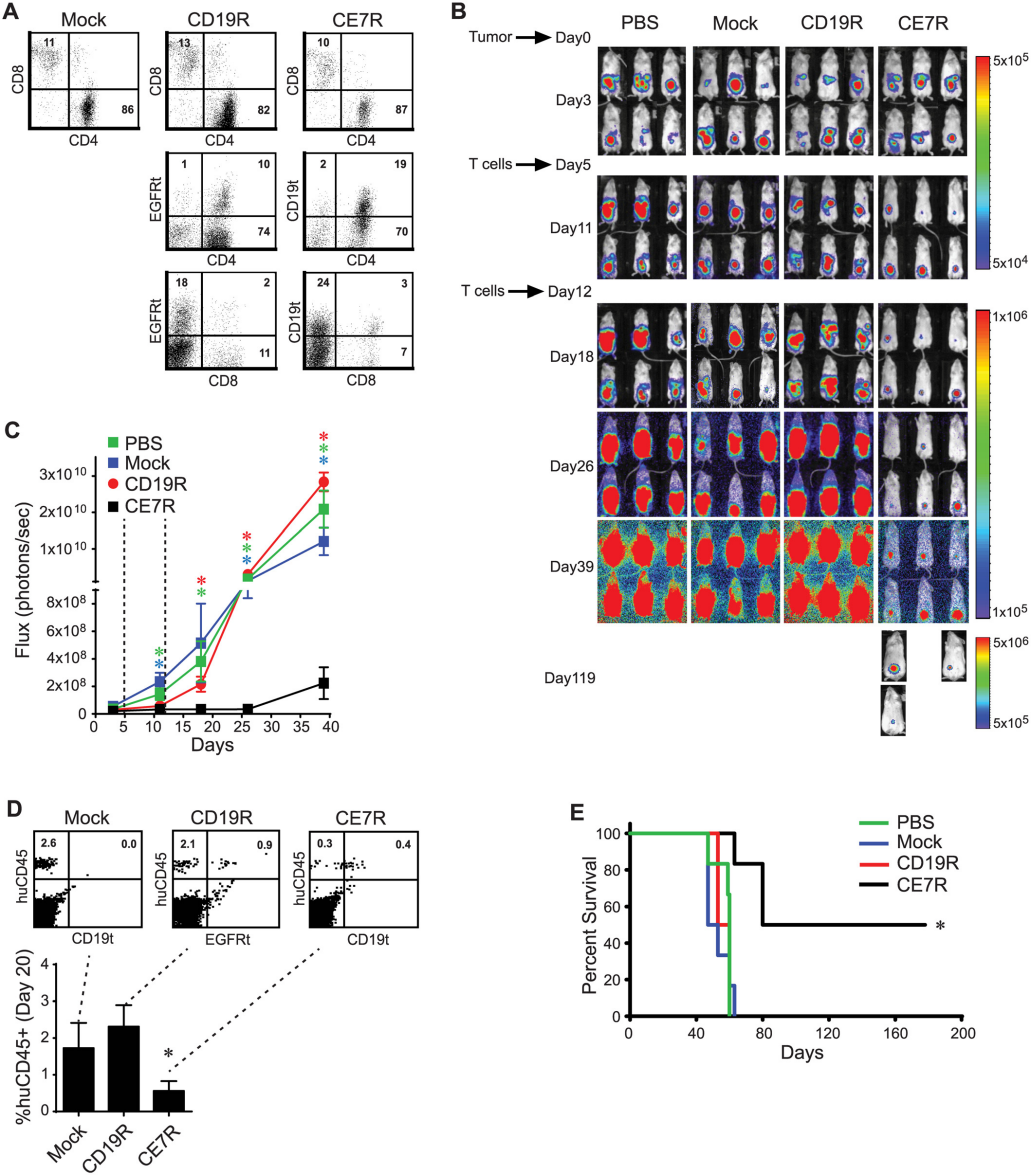
CE7R^+^ T_CM_ Cells Inhibit Intraperitoneal Ovarian Tumor Growth and Ascites Formation *in Vivo*. **A**, Flow cytometric analysis of mock-, CE7R- and CD19R-transduced T_CM_ cells prior to use *in vivo*. Percentages of positive cells in each quadrant are indicated. **B**, NSG mice received i.p. injection of ffLuc+ SK-OV-3 tumor cells on day 0, and were randomized into 4 groups (n = 6 mice per group) for treatment with two doses of either PBS or mock-, CE7R- or CD19R-transduced T cells i.p. (i.e, days 5 and 12). Results are representative of three independent experiments. **C**, Quantitative bioluminescence imaging of for each group over the time. Mean ± S.E. of total flux levels of luciferase activity were measured. Dashed lines represent days of T cell treatment. *, p < 0.05 when comparing flux values at the indicated timepoints using the unpaired Student’s t-test. **D**, Flow cytometric detection of T cells in peripheral blood 8 days after the second dose of T cells were administered. Representative histograms and percentages of human CD45^+^ cells (mean + S.D.) are depicted for each group of mice. *, p < 0.003 when the CE7R^+^ T cell group was compared to either the Mock or CD19R^+^ T cell treated groups. **E**, Kaplan-Meier analysis of survival for each group. *, p ≤ 0.001 when the CE7R^+^ T cell treated group was compared to any other group using the Log-rang (Mantel-Cox) test.

Primary human T_CM_ cells that were bead stimulated and then mock-transduced, or lentivirally transduced to express either the CE7R or an unrelated control, CD19-specific CAR (CD19R), were characterized for their phenotype prior to their use in mice (Fig 3A). Of note, both CAR sequences in these studies contained the L235E/N297Q dual mutation within their Fc spacer that we have shown to reduce binding to soluble FcγRs and increase *in vivo* persistence without altering the ability of the CAR to mediate target antigen-specific lysis [20]. Overall, the mock-, CD19R-, and CE7R-transduced T cells were similar in terms of their percentages of CD4^+^ (82–87%) and CD8^+^ (10–13%) populations. T cell doses given to mice were then normalized based on CAR positivity, so that any tumor response differences would not be attributed to differences in transduction efficiency between the T cell lines. Thus, after confirming ffLuc+ SK-OV-3 tumor establishment by bioluminescence imaging, mice received two i.p. doses of either 5 x 10^6^ CAR^+^ T cells, mock-transduced T cells, or PBS, at day 5 and at day 12 after tumor cell administration. Control mice (PBS, mock, or CD19R treated) all exhibited progression of i.p. ovarian tumor growth, became moribund and had to be euthanized within 2 months. In contrast, treatment with the CE7R^+^ T cells significantly inhibited tumor growth (Fig 3B and 3C). Six months after treatment with CE7R^**+**^ T cells, 50% (3 of 6) mice remained alive without signs of distress, and imaging at day 119 post-tumor cell injection showed that two mice still had only minimum tumor signal (Fig 3B).

Interestingly, levels of human T cells (huCD45^+^) in the peripheral blood 8 days after the last T cell administration (day 20 post tumor cell injection) were low but consistently detectable in the mock, CD19R^+^ and CE7R^+^ T cell-treated groups (Fig 3D), with the least amount of huCD45^+^ cells in the CE7R^+^ T cell treated group. We hypothesize that the majority of i.p. administered L1-CAM-specific CAR T cells may have remained in the peritoneal cavity—which is in line with our observations in other experiments where CE7R^+^ T cells were most easily detected in peritoneal washes (and not in peripheral blood) of OVCAR-3 tumor bearing mice up to three weeks post i.p. administration (S1 Fig). Regardless of T cell peripheral persistence and/or localization, the CE7R^+^ T cell-treated, mice exhibited significantly improved survival (median survival of 104.5 days) in comparison to PBS (median survival of 60 days, p = 0.0012), mock (median survival of 50 days, P = 0.001), and CD19R (median survival of 56.5 days, p = 0.0009) treated controls (Fig 3E).

### Residual tumors post CE7R^+^ T cell therapy exhibit decreased ascites and reduced L1-CAM expression

Upon autopsy of euthanized mice in each group, it was found that 60% of the mice in the control groups (Table 1) had distended abdomens and various amounts of bloody ascites (as much as 3mL per mouse). In striking contrast, mice which developed recurrent tumors post CE7R^+^ T cell treatment, evaluated once they became symptomatic, did not exhibit prominent distension of the abdomen, nor did they have any bloody ascites detectable upon autopsy (Fig 4A). Furthermore, when collected tumor nodules were subjected to immuohistochemical staining with CE7 antibody, tumors from the control mice (PBS, Mock and CD19R treated) displayed strong, albeit heterogenous, L1-CAM/CE7 staining (Fig 4B). Residual tumor collected from CE7R^+^ T cell-treated mice, however, displayed substantially decreased L1-CAM/CE7 expression in terms of both percentages of positive cells and overall intensity of staining. This data suggests that CE7R^**+**^ T cells may have specifically eliminated the SK-OV-3 tumor cells expressing higher levels of L1-CAM antigen, while allowing residual L1-CAM low/negative subpopulations to remain. Further, this experiment also suggests that lower L1-CAM expression correlates with decreased ascites production by the i.p. engrafted tumors.

**Table 1:** Ascites Formation in Each Group of Mice.

	# mice with ascites/# total mice
**PBS**	3/6
**Mock**	4/6
**CD19R**	4/6
**CE7R**	0/6

**Fig 4:**
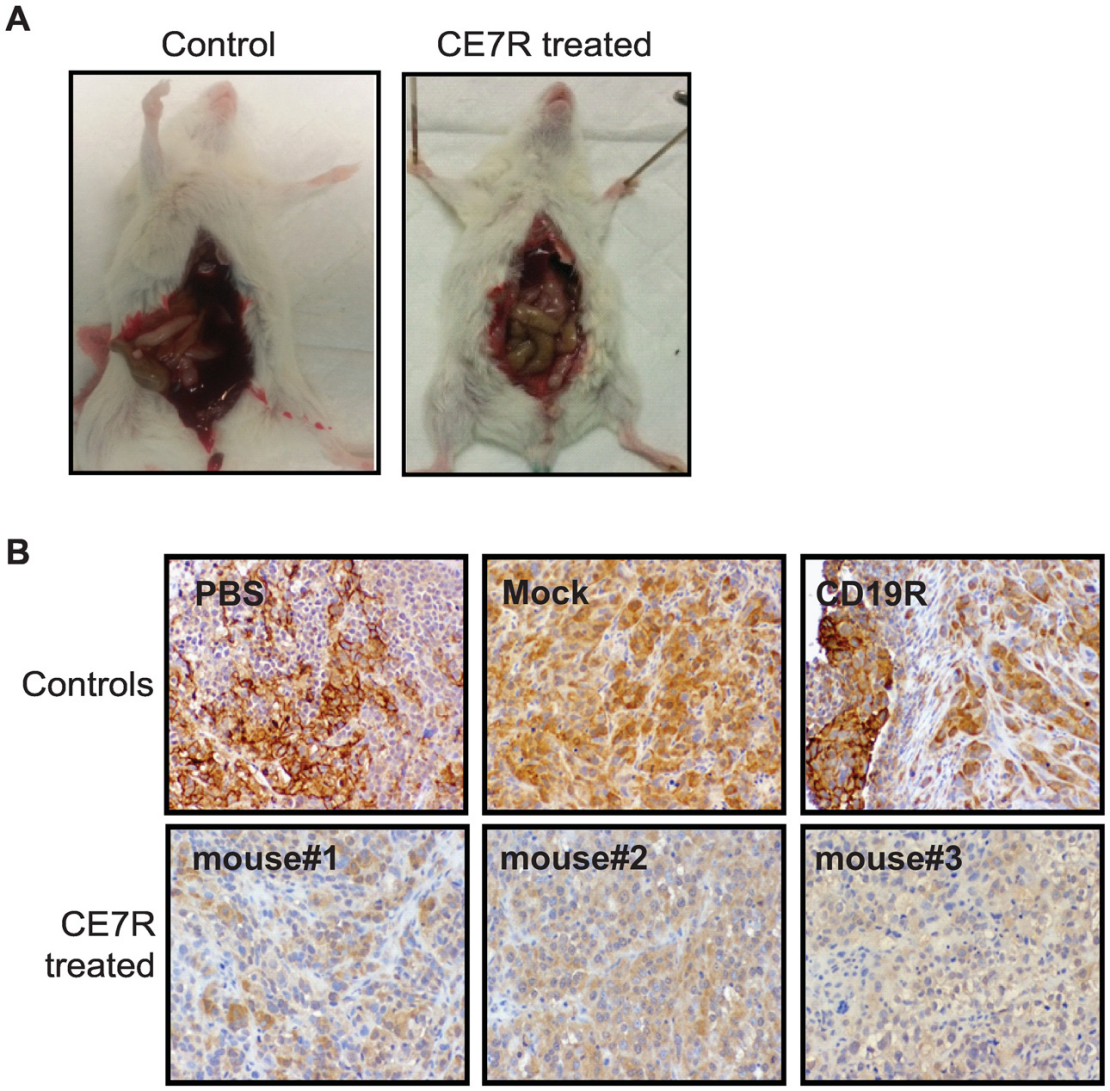
Treatment with CE7R^+^ T Cells Results in Less Ascites Rormation and Reduced L1-CAM Expression on Residual Tumors. **A**, Representative images of ascites formation in control mice (PBS, mock or CD19R^+^ T cell treated groups) versus CE7R^+^ T cell treated mice. **B**, Tumor nodules were resected from each mouse upon euthanasia when mice became moribund and subjected to immunohistochemical staining via CE7 monoclonal antibody. Images of representative tumors from PBS, mock-transduced T cell (Mock), CD19R^+^ T cell (CD19R) or CE7R^+^ T cell (mouse#1, #2, #3) treated mice are depicted. Photomicrographs are shown at magnification of x200 (ocular 10x; objective 20x).

### CE7R^+^ T cells exhibit *in vitro* effector activity against primary ovarian cancer cells

**Fig 5:**
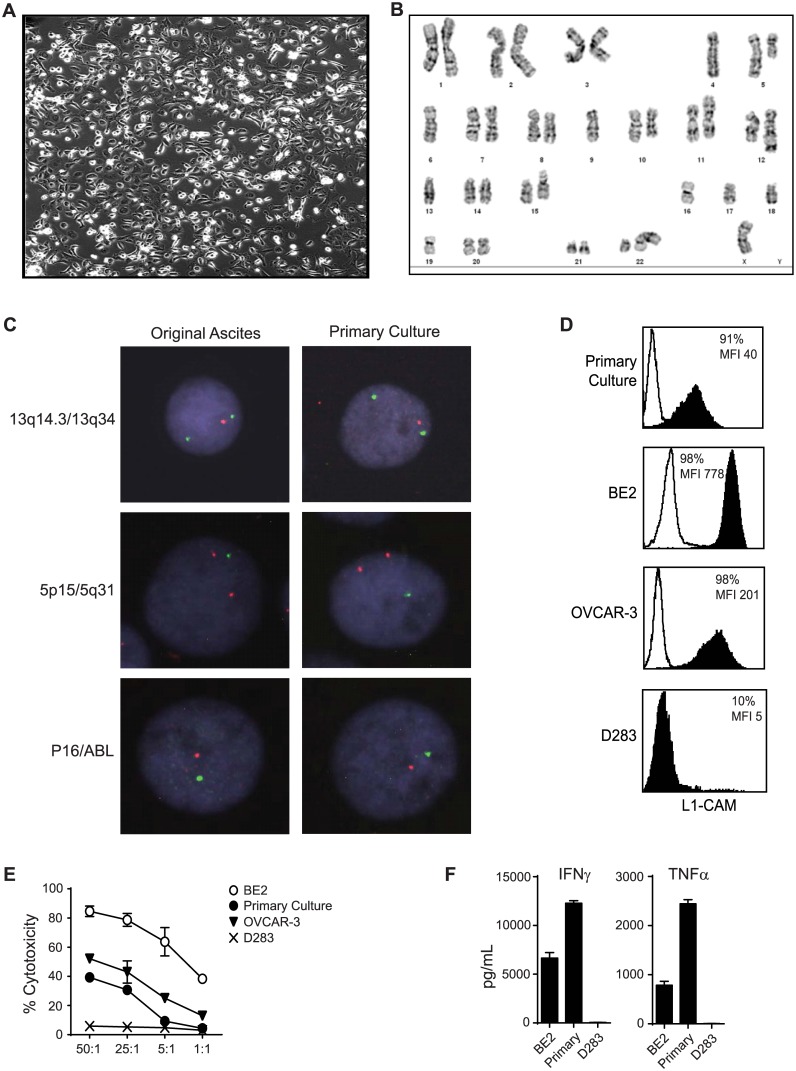
CE7R^+^ T Cells Specifically Recognized and Killed L1-CAM-Expressing Primary Ovarian Cancer Cells Derived from Malignant Ascites. **A**, Representative primary culture of ovarian cancer cells derived from patient ascites depicting typical epithelial cobblestone morphology. **B**, Representative cytogenetic analysis of primary culture of ovarian cancer cells derived from patient ascites. Stemline: 37, X, -X, der(2)t(2;6)(q35;p11), -4, del(5)(q13q33), -6, del(8)(p21p23), 9, del(10) (q24q26), der(11)t(11;?15) (p15;q15), der(12)t(12;?13) (q24.3;q22), -13, -16, -17, -18, 19, der(22)t(12;22)(q11;p13). **C**, Representative images of fluorescence *in situ* hybridization for cultured primary ovarian cancer cells in comparison to original ascites cells using probes for 13q14.3 (red) and 13q34 (green), 5p15 (red) and 5q31 (green), or P16 (red) and ABL (green). **D**, Flow cytometric examination of cell-surface L1-CAM expression on the primary cultured, ascites-derived cancer cells compared to the BE2 neuroblastoma, OVCAR-3 ovarian cancer, and D283 medulloblastoma cell lines. Mean fluorescent intensity (MFI) and percentages of cells with positive staining (%) over secondary reagent alone are indicated. **E**, CE7R^+^ T cells were co-cultured overnight with the indicated tumor lines at a 10:1 ratio and supernatants were analyzed for IFN-γ and TNF-α levels by cytometric bead array. Means + S.E.M. of triplicate wells are depicted. **F**, CE7R^+^ T cells against the indicated cancer cell lines targets was determined by 4-hr ^51^Cr-release assay. Mean % chromium release ± SD of triplicate wells are depicted.

After demonstrating proof-of-principle therapy with long-term established, commercially available ovarian cancer cell lines, we next sought to investigate whether genetically redirected CE7R^+^ T cells have the ability to target and kill primary human ovarian tumor cells derived from patients. Such primary ovarian cancer cells are thought to be a more clinically relevant for characterizing the potential efficacy of novel therapeutic strategies. As a result, we developed a protocol to establish primary cultures of ovarian cancer cells from the fresh ascites fluid of ovarian cancer patients. In ascites, there are some potential contaminating non-malignant cells present in addition to cancer cells, such as stromal fibroblasts and endothelial cells. Indeed, in our hands, fibroblast contamination of primary cultures isolated directly from patient ascites fluid was often observed, and these cells quickly outgrew the ovarian cancer cells. Therefore once primary lines were established, we confirmed the malignant nature of these cells in culture. Several evaluation criteria were utilized. First, cells growing in tissue culture were examined by phase contrast microscopy and only cultured cells that exhibited as a monolayer cobblestone-like appearance ascribed to epithelium *in vitro* were used for all subsequent studies (Fig 5A). Cultures with significant portions of fibroblast-like morphology, which suggested the outgrowth of contaminating stromal fibroblasts, were not used for further experimentation. Second, conventional cytogenetic analysis was performed to determine aberrations in both chromosomal number and structure in cultured primary cells, which would confirm that these cells were indeed transformed. For example, a representative karyotype (Fig 5B) showed that eighteen pairs of chromosomes had numerical and/or structural abnormalities. The hypodiploid stemline was characterized by structural rearrangements involving chromosomes 2, 5, 6, 8, 10, 11, 12, 13, 15, and 22, as well as clonal losses of chromosomes X, 4, 6, 9, 13, 16, 17, 18, and 19. Although the karyotype is hypodiploid, two structural rearrangements result in trisomy for the long arm of chromosome 12 and partial trisomy for the long arm of chromsome 15. Furthermore, a hypotetraploid side-line contained duplicate copies of the stemline abnormalities, and small second sideline of two cells contained an interstitial deletion of the long arm of chromosome 2, as well as the stemline aberrations. Third, to confirm that the genetic aberrations observed in cultured primary cells were truly representative of the original cancer cells in ascites, and not an artifact acquired during extended periods of *ex vivo* culture, we closely compared the chromosomal abnormalities in our cultured primary cells with the original ascitic cells by FISH. The results showed excellent correlation of signal patterns at all nine interrogated loci providing strong evidence that the cultured cells were derived from the original tumor and maintained the cytogenetic abnormalities of the original tumor (Fig 5C).

Importantly, the primary ascetic cells that were positive for L1-CAM (Fig 1C) remained L1-CAM positive after *ex vivo* culture (Fig 5D), and CE7R^**+**^ T cells efficiently lysed the L1-CAM/CE7-positive, primary cultured ovarian cancer cells (Fig 5E). Furthermore, co-culture of these cells with CE7R^**+**^ T cells resulted in the T cells secreting considerable amounts of pro-inflammatory cytokines such as IFN-γ and TNF-α (Fig 5F) in an antigen dependent manner. These data suggest that primary ovarian cancer cells are amenable to targeting by CAR-redirected T cells.

## Discussion

To date, CAR-based T cell therapy using genetically re-directed primary human lymphocytes to target tumor-associated antigens has shown promising responses in clinical trials, particularly in the setting of CD19-positive B-cell lineage malignancies (reviewed in [4, 5]). CAR-modified T cells not only exert immediate effector functions but also have the potential to act as “living drugs” to establish long-term immunity. However, CAR-expressing T cells have additional hurdles in the solid tumor setting, most notably due to the general lack of tumor antigen specificity [35]. L1-CAM has been previously documented to be over-expressed in many solid tumors including ovarian cancer [12, 36–39]. We report here that the CE7-epitope of L1-CAM is a clinically relevant ovarian cancer-associated antigen as it is absent on normal ovaries, but highly expressed on established ovarian cancer cell lines, primary ovarian cancer tissues regardless of histological subtypes, and ovarian cancer cells derived from patient ascites.

L1-CAM exhibits various properties that provide rationale for targeting it in cancer therapies. For example, L1-CAM has been shown in pancreatic and ovarian carcinoma cells to augment protection from apoptosis and to contribute to chemoresistance [40, 41]. L1-CAM has also been implicated in promoting tumor cell proliferation, as well as migration, invasion and metastasis [16, 42]. Furthermore, the presence of L1-CAM in cancer tissue has been found to correlate with more advanced stages of disease and poor prognosis [11, 13, 43, 44]. Taken together, these findings have stimulated interest in targeting L1-CAM in cancer using either antibody-based or radioimmunotherapy-based therapeutic approaches [16, 45]. We have also examined the utility of genetically engineered primary human T cells with a CAR directed against the CE7 epitope of L1-CAM for the treatment of neuroblastoma [17, 19]. Here we extend this work to ovarian cancer, using a second generation CD28-containing CAR and a clinically translatable manufacturing platform using enriched central memory T cells (T_CM_) for genetic modification. Importantly, these CE7R^+^ T_CM_ cells were capable of secreting high levels of IFN-γ and TNF-α, and exhibited robust and specific *in vitro* lytic activity against L1-CAM/CE7^+^ ovarian tumor cells—both established lines and primary tumor cells derived from the malignant ascites of ovarian cancer patients.

Interestingly, our data suggest that relatively low tumor antigen density (e.g., as seen on MADH2744 cells) can be sufficient to trigger the effector activity of CE7R^+^ T cells. This may be particularly relevant to targeting L1-CAM in ovarian cancer, which, like most solid tumors, appears to exhibit heterogenous antigen expression both within and between patient tumors. Additional studies evaluating CAR design, including varying linker length, intracellular costimatory domains (i.e. CD28, 4-1BB), and even alternative scFv to target L1-CAM may be important in optimizing the CAR for targeting higher antigen density. Indeed, the choice of extracellular spacer in combination with cytoplasmic signaling modules has recently been found to be critical in ‘fine tuning’ the *in vivo* anti-tumor potency of CE7-specific CARs [46]. As L1-CAM is known to be expressed on several normal tissues including the stomach, kidney, adrenal glands, and in certain regions of the nervous system [12], these future studies are also warranted to address potential on-target off-tumor toxicity of L1-CAM-specific CAR T cells. Alternatively, there are additional strategies to obviate the possible toxicities associated with the presence of L1-CAM in normal tissues when using CAR-based T cell therapy in treating cancer, including the use of mRNA transfection-based gene transfer approaches rather than a lentiviral delivery method for CAR introduction in T cells [47], as well as incorporation of a suicide gene strategy such as EGFRt [26] or inducible caspase-9 [48], to selectively eliminate CAR T cells in patients should adverse events occur.

We have shown here that L1-CAM-specific CAR T cells provide significant growth control of solid tumors in an *in vivo* ovarian cancer xenograft model that exhibited the clinically relevant features of widespread cancerous matastases in the peritoneal cavity and massive ascites. Ovarian tumor growth in this model was significantly delayed and mouse survival was extended by administration of two doses of CE7R^+^ T_CM_. Notably, the anti-tumor effect was antigen-specific, as adoptive transfer of CD19-specific CAR-expressing T_CM_ had no impact on tumor progression and survival. Ascites formation was also dramatically inhibited in CE7R^+^ T_CM_-treated mice, while over half of the control mice (11/18) developed massive bloody ascites. Of note, while the CE7R^+^ T cells did not completely eradicate SK-OV-3 tumor growth in our *in vivo* study, the recurrent/residual tumors had greatly reduced L1-CAM/CE7 antigen expression, suggesting antigen ‘loss’ as a potential tumor escape mechanism. This has led us to consider future examination of combinatorial strategies, such as a multiple-targeting CAR approach (i.e., to target more than one tumor associated antigen) [49].

Currently, combination chemotherapies have not substantially decreased the mortality rate in ovarian cancer [50]. Unlike conventional chemotherapeutics and antibody-based therapies, CAR-redirected T lymphocytes have the potential to proliferate and persist in humans, and establish long-term anti-tumor immunity. This, together with the pre-clinical data presented here, supports the further development of an L1-CAM-specific, CAR-based adoptive therapy approach for the treatment of ovarian cancer.

## Supporting Information

S1 Fig CE7R+ T Cells are Detected in the Peritoneal Wash of Intraperitoneal L1-CAM+ Tumor Bearing Mice. NSG mice recived i.p. injection of 10e5 ffLuc+ OVCAR-3 tumor cells on day 0, and were treated with asingle dose of 2.5 x 10e7 CE7R-transduced T cells i.p. (day 7). Flow cytometric detection of human T cells in the peripheral blood at day 22 (A) and in the peritoneal wash upon euthanasia (B) of representative mice (Mouse 1 and Mouse 2) at either day 22 or day 30 as indicated. Percentages of human CD45+ cells (A, B), or human CD45-gated cells that were stained with biotinylated anti-Fcγ followed by SA-PE to detect the CAR (B), are indicated in each histogram. C, Quantitative bioluminescence imaging of tumor growth in Mouse 1 (top) and Mouse 2 (bottom) over time. Mean flux levels of luciferase activity were measured. Dashed lines represent day of T cell treatment.
(PDF)

## References

[1] Siegel R, Naishadham D, Jemal A. Cancer statistics, 2013. CA Cancer J Clin. 2013 1;63(1):11–30. 10.3322/caac.21166 23335087

[2] Dinh P, Harnett P, Piccart-Gebhart MJ, Awada A. New therapies for ovarian cancer: cytotoxics and molecularly targeted agents. Crit Rev Oncol Hematol. 2008 8;67(2):103–12. 10.1016/j.critrevonc.2008.01.012 18342536

[3] Wefers C, Lambert LJ, Torensma R, Hato SV. Cellular immunotherapy in ovarian cancer: Targeting the stem of recurrence. Gynecol Oncol. 2015 2 26. 10.1016/j.ygyno.2015.02.019 25727651

[4] Ramos CA, Savoldo B, Dotti G. CD19-CAR trials. Cancer J. 2014 Mar-Apr;20(2):112–8. 24667955 10.1097/PPO.0000000000000031 PMC3979594

[5] Maus MV, Grupp SA, Porter DL, June CH. Antibody-modified T cells: CARs take the front seat for hematologic malignancies. Blood. 2014 4 24;123(17):2625–35. 10.1182/blood-2013-11-492231 24578504 PMC3999751

[6] Cheadle EJ, Gornall H, Baldan V, Hanson V, Hawkins RE, Gilham DE. CAR T cells: driving the road from the laboratory to the clinic. Immunol Rev. 2014 1;257(1):91–106. 10.1111/imr.12126 24329792

[7] Andersson E, Villabona L, Bergfeldt K, Carlson JW, Ferrone S, Kiessling R, et al Correlation of HLA-A02* genotype and HLA class I antigen down-regulation with the prognosis of epithelial ovarian cancer. Cancer Immunol Immunother. 2012 8;61(8):1243–53. 10.1007/s00262-012-1201-0 22258792 PMC8693725

[8] Lanitis E, Poussin M, Klattenhoff AW, Song D, Sandaltzopoulos R, June CH, et al Chimeric antigen receptor T Cells with dissociated signaling domains exhibit focused antitumor activity with reduced potential for toxicity in vivo. Cancer immunology research. 2013 7;1(1):43–53. 24409448 10.1158/2326-6066.CIR-13-0008 PMC3881605

[9] Chekmasova AA, Rao TD, Nikhamin Y, Park KJ, Levine DA, Spriggs DR, et al Successful eradication of established peritoneal ovarian tumors in SCID-Beige mice following adoptive transfer of T cells genetically targeted to the MUC16 antigen. Clin Cancer Res. 2010 7 15;16(14):3594–606. 10.1158/1078-0432.CCR-10-0192 20628030 PMC2907178

[10] Kershaw MH, Westwood JA, Parker LL, Wang G, Eshhar Z, Mavroukakis SA, et al A phase I study on adoptive immunotherapy using gene-modified T cells for ovarian cancer. Clin Cancer Res. 2006 10 15;12(20 Pt 1):6106–15. 17062687 10.1158/1078-0432.CCR-06-1183 PMC2154351

[11] Daponte A, Kostopoulou E, Kollia P, Papamichali R, Vanakara P, Hadjichristodoulou C, et al L1 (CAM) (CD171) in ovarian serous neoplasms. Eur J Gynaecol Oncol. 2008;29(1):26–30. 18386459

[12] Hong H, Stastny M, Brown C, Chang WC, Ostberg JR, Forman SJ, et al Diverse Solid Tumors Expressing a Restricted Epitope of L1-CAM Can Be Targeted by Chimeric Antigen Receptor Redirected T Lymphocytes. J Immunother. 2014 Feb-Mar;37(2):93–104. 24509172 10.1097/CJI.0000000000000018

[13] Boo YJ, Park JM, Kim J, Chae YS, Min BW, Um JW, et al L1 expression as a marker for poor prognosis, tumor progression, and short survival in patients with colorectal cancer. Ann Surg Oncol. 2007 5;14(5):1703–11. 17211730 10.1245/s10434-006-9281-8

[14] Gavert N, Ben-Shmuel A, Raveh S, Ben-Ze'ev A. L1-CAM in cancerous tissues. Expert Opin Biol Ther. 2008 11;8(11):1749–57. 10.1517/14712598.8.11.1749 18847309

[15] Schroder C, Schumacher U, Fogel M, Feuerhake F, Muller V, Wirtz RM, et al Expression and prognostic value of L1-CAM in breast cancer. Oncol Rep. 2009 11;22(5):1109–17. 19787228 10.3892/or_00000543

[16] Arlt MJ, Novak-Hofer I, Gast D, Gschwend V, Moldenhauer G, Grunberg J, et al Efficient inhibition of intra-peritoneal tumor growth and dissemination of human ovarian carcinoma cells in nude mice by anti-L1-cell adhesion molecule monoclonal antibody treatment. Cancer Res. 2006 1 15;66(2):936–43. 16424028 10.1158/0008-5472.CAN-05-1818

[17] Park JR, Digiusto DL, Slovak M, Wright C, Naranjo A, Wagner J, et al Adoptive transfer of chimeric antigen receptor re-directed cytolytic T lymphocyte clones in patients with neuroblastoma. Mol Ther. 2007 4;15(4):825–33. 17299405 10.1038/sj.mt.6300104

[18] Wang X, Berger C, Wong CW, Forman SJ, Riddell SR, Jensen MC. Engraftment of human central memory-derived effector CD8+ T cells in immunodeficient mice. Blood. 2011 2 10;117(6):1888–98. 10.1182/blood-2010-10-310599 21123821 PMC3056638

[19] Gonzalez S, Naranjo A, Serrano LM, Chang WC, Wright CL, Jensen MC. Genetic engineering of cytolytic T lymphocytes for adoptive T-cell therapy of neuroblastoma. Journal of Gene Medicine. 2004;6(6):704–11. 15170741 10.1002/jgm.489

[20] Jonnalagadda M, Mardiros A, Urak R, Wang X, Hoffman LJ, Bernanke A, et al Chimeric Antigen Receptors With Mutated IgG4 Fc Spacer Avoid Fc Receptor Binding and Improve T Cell Persistence and Antitumor Efficacy. Mol Ther. 2015 4;23(4):757–68. 10.1038/mt.2014.208 25366031 PMC4395772

[21] Nguyen P, Moisini I, Geiger TL. Identification of a murine CD28 dileucine motif that suppresses single-chain chimeric T-cell receptor expression and function. Blood. 2003 12 15;102(13):4320–5. 12946999 10.1182/blood-2003-04-1255

[22] Kowolik CM, Topp MS, Gonzalez S, Pfeiffer T, Olivares S, Gonzalez N, et al CD28 costimulation provided through a CD19-specific chimeric antigen receptor enhances in vivo persistence and antitumor efficacy of adoptively transferred T cells. Cancer Res. 2006 11 15;66(22):10995–1004. 17108138 10.1158/0008-5472.CAN-06-0160

[23] Szymczak AL, Workman CJ, Wang Y, Vignali KM, Dilioglou S, Vanin EF, et al Correction of multi-gene deficiency in vivo using a single 'self-cleaving' 2A peptide-based retroviral vector. Nat Biotechnol. 2004 5;22(5):589–94. 15064769 10.1038/nbt957

[24] Jonnalagadda M, Brown CE, Chang WC, Ostberg JR, Forman SJ, Jensen MC. Efficient selection of genetically modified human T cells using methotrexate-resistant human dihydrofolate reductase. Gene Ther. 2013 1 10;20(8):853–60. 10.1038/gt.2012.97 23303282 PMC4028078

[25] Cooper LJ, Topp MS, Serrano LM, Gonzalez S, Chang WC, Naranjo A, et al T-cell clones can be rendered specific for CD19: toward the selective augmentation of the graft-versus-B-lineage leukemia effect. Blood. 2003 2 15;101(4):1637–44. 12393484 10.1182/blood-2002-07-1989

[26] Wang X, Chang WC, Wong CW, Colcher D, Sherman M, Ostberg JR, et al A transgene-encoded cell surface polypeptide for selection, in vivo tracking, and ablation of engineered cells. Blood. 2011 8 4;118(5):1255–63. 10.1182/blood-2011-02-337360 21653320 PMC3152493

[27] Wang X, Naranjo A, Brown CE, Bautista C, Wong CW, Chang WC, et al Phenotypic and Functional Attributes of Lentivirus-modified CD19-specific Human CD8+ Central Memory T Cells Manufactured at Clinical Scale. J Immunother. 2012 11;35(9):689–701. 23090078 10.1097/CJI.0b013e318270dec7 PMC3525345

[28] Kahlon KS, Brown C, Cooper LJ, Raubitschek A, Forman SJ, Jensen MC. Specific recognition and killing of glioblastoma multiforme by interleukin 13-zetakine redirected cytolytic T cells. Cancer Res. 2004 12 15;64(24):9160–6. 15604287 10.1158/0008-5472.CAN-04-0454

[29] Zecchini S, Bianchi M, Colombo N, Fasani R, Goisis G, Casadio C, et al The differential role of L1 in ovarian carcinoma and normal ovarian surface epithelium. Cancer Res. 2008 2 15;68(4):1110–8. 10.1158/0008-5472.CAN-07-2897 18281486

[30] Bondong S, Kiefel H, Hielscher T, Zeimet AG, Zeillinger R, Pils D, et al Prognostic significance of L1CAM in ovarian cancer and its role in constitutive NF-kappaB activation. Ann Oncol. 2012 7;23(7):1795–802. 10.1093/annonc/mdr568 22228447

[31] Brentjens RJ, Santos E, Nikhamin Y, Yeh R, Matsushita M, La Perle K, et al Genetically targeted T cells eradicate systemic acute lymphoblastic leukemia xenografts. Clin Cancer Res. 2007 9 15;13(18 Pt 1):5426–35. 17855649 10.1158/1078-0432.CCR-07-0674

[32] Zhong XS, Matsushita M, Plotkin J, Riviere I, Sadelain M. Chimeric antigen receptors combining 4-1BB and CD28 signaling domains augment PI3kinase/AKT/Bcl-XL activation and CD8+ T cell-mediated tumor eradication. Mol Ther. 2010 2;18(2):413–20. 10.1038/mt.2009.210 19773745 PMC2839303

[33] Berger C, Jensen MC, Lansdorp PM, Gough M, Elliott C, Riddell SR. Adoptive transfer of effector CD8 T cells derived from central memory cells establishes persistent T cell memory in primates. J Clin Invest. 2008 1 2;118(1):294–305. 18060041 10.1172/JCI32103 PMC2104476

[34] Graef P, Buchholz VR, Stemberger C, Flossdorf M, Henkel L, Schiemann M, et al Serial transfer of single-cell-derived immunocompetence reveals stemness of CD8(+) central memory T cells. Immunity. 2014 7 17;41(1):116–26. 10.1016/j.immuni.2014.05.018 25035956

[35] Kakarla S, Gottschalk S. CAR T Cells for Solid Tumors: Armed and Ready to Go? Cancer J. 2014 Mar-Apr;20(2):151–5. 24667962 10.1097/PPO.0000000000000032 PMC4050065

[36] Huszar M, Moldenhauer G, Gschwend V, Ben-Arie A, Altevogt P, Fogel M. Expression profile analysis in multiple human tumors identifies L1 (CD171) as a molecular marker for differential diagnosis and targeted therapy. Hum Pathol. 2006 8;37(8):1000–8. 16867862 10.1016/j.humpath.2006.03.014

[37] Gavert N, Conacci-Sorrell M, Gast D, Schneider A, Altevogt P, Brabletz T, et al L1, a novel target of beta-catenin signaling, transforms cells and is expressed at the invasive front of colon cancers. J Cell Biol. 2005 2 14;168(4):633–42. 15716380 10.1083/jcb.200408051 PMC2171754

[38] Kaifi JT, Zinnkann U, Yekebas EF, Schurr PG, Reichelt U, Wachowiak R, et al L1 is a potential marker for poorly-differentiated pancreatic neuroendocrine carcinoma. World J Gastroenterol. 2006 1 7;12(1):94–8. 16440424 10.3748/wjg.v12.i1.94 PMC4077503

[39] Allory Y, Matsuoka Y, Bazille C, Christensen EI, Ronco P, Debiec H. The L1 cell adhesion molecule is induced in renal cancer cells and correlates with metastasis in clear cell carcinomas. Clin Cancer Res. 2005 2 1;11(3):1190–7. 15709188

[40] Sebens Muerkoster S, Werbing V, Sipos B, Debus MA, Witt M, Grossmann M, et al Drug-induced expression of the cellular adhesion molecule L1CAM confers anti-apoptotic protection and chemoresistance in pancreatic ductal adenocarcinoma cells. Oncogene. 2007 4 26;26(19):2759–68. 17086212 10.1038/sj.onc.1210076

[41] Stoeck A, Gast D, Sanderson MP, Issa Y, Gutwein P, Altevogt P. L1-CAM in a membrane-bound or soluble form augments protection from apoptosis in ovarian carcinoma cells. Gynecol Oncol. 2007 2;104(2):461–9. 17030349 10.1016/j.ygyno.2006.08.038

[42] Gast D, Riedle S, Issa Y, Pfeifer M, Beckhove P, Sanderson MP, et al The cytoplasmic part of L1-CAM controls growth and gene expression in human tumors that is reversed by therapeutic antibodies. Oncogene. 2008 2 21;27(9):1281–9. 17952127 10.1038/sj.onc.1210747

[43] Doberstein K, Wieland A, Lee SB, Blaheta RA, Wedel S, Moch H, et al L1-CAM expression in ccRCC correlates with shorter patients survival times and confers chemoresistance in renal cell carcinoma cells. Carcinogenesis. 2011 3;32(3):262–70. 10.1093/carcin/bgq249 21097529

[44] Li S, Jo YS, Lee JH, Min JK, Lee ES, Park T, et al L1 cell adhesion molecule is a novel independent poor prognostic factor of extrahepatic cholangiocarcinoma. Clin Cancer Res. 2009 12 1;15(23):7345–51. 10.1158/1078-0432.CCR-09-0959 19920102

[45] Fischer E, Grunberg J, Cohrs S, Hohn A, Waldner-Knogler K, Jeger S, et al L1-CAM-targeted antibody therapy and (177)Lu-radioimmunotherapy of disseminated ovarian cancer. Int J Cancer. 2012 6 1;130(11):2715–21. 10.1002/ijc.26321 21796623

[46] Kunkele A, Johnson AJ, Rolczynski LS, Chang CA, Hoglund V, Kelly-Spratt KS, et al Functional Tuning of CARs Reveals Signaling Threshold above Which CD8+ CTL Antitumor Potency Is Attenuated due to Cell Fas-FasL-Dependent AICD. Cancer immunology research. 2015 4;3(4):368–79. 10.1158/2326-6066.CIR-14-0200 25576337

[47] Zhao Y, Moon E, Carpenito C, Paulos CM, Liu X, Brennan AL, et al Multiple injections of electroporated autologous T cells expressing a chimeric antigen receptor mediate regression of human disseminated tumor. Cancer Res. 2010 11 15;70(22):9053–61. 10.1158/0008-5472.CAN-10-2880 20926399 PMC2982929

[48] Di Stasi A, Tey SK, Dotti G, Fujita Y, Kennedy-Nasser A, Martinez C, et al Inducible apoptosis as a safety switch for adoptive cell therapy. N Engl J Med. 2011 11 3;365(18):1673–83. 10.1056/NEJMoa1106152 22047558 PMC3236370

[49] Hegde M, Corder A, Chow KK, Mukherjee M, Ashoori A, Kew Y, et al Combinational targeting offsets antigen escape and enhances effector functions of adoptively transferred T cells in glioblastoma. Mol Ther. 2013 11;21(11):2087–101. 10.1038/mt.2013.185 23939024 PMC3831041

[50] Jayson GC, Kohn EC, Kitchener HC, Ledermann JA. Ovarian cancer. Lancet. 2014 10 11;384(9951):1376–88. 10.1016/S0140-6736(13)62146-7 24767708

