# Supplementary figures and images for: L1 Cell Adhesion Molecule-Specific Chimeric Antigen Receptor-Redirected Human T Cells Exhibit Specific and Efficient Antitumor Activity against Human Ovarian Cancer in Mice
Source: PLoS One. 2016 Jan 13;11(1):e0146885. doi: 10.1371/journal.pone.0146885 (PMC4711972; doi:10.1371/journal.pone.0146885)

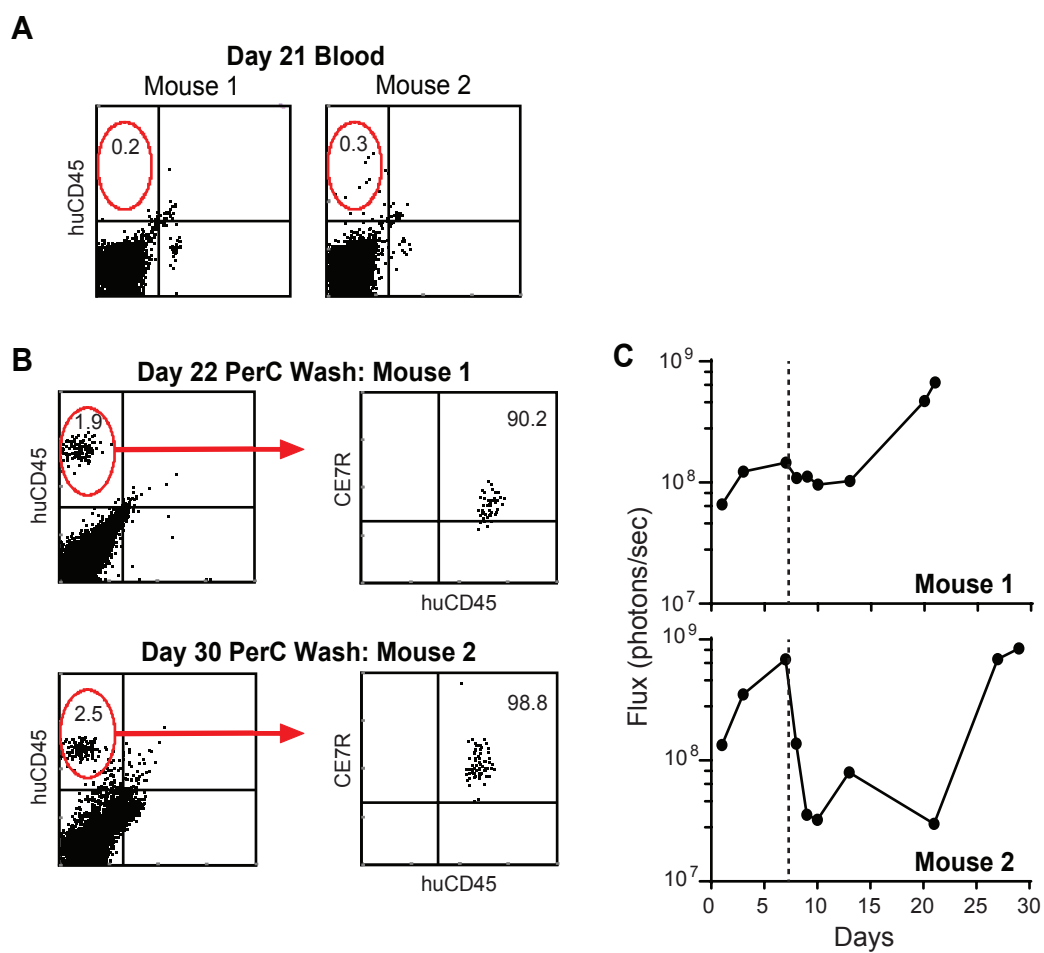

Supplement: S1 Fig — NSG mice recived i.p. injection of 10e5 ffLuc+ OVCAR-3 tumor cells on day 0, and were treated with asingle dose of 2.5 x 10e7 CE7R-transduced T cells i.p. (day 7). Flow cytometric detection of human T cells in the peripheral blood at day 22 (A) and in the peritoneal wash upon euthanasia (B) of representative mice (Mouse 1 and Mouse 2) at either day 22 or day 30 as indicated. Percentages of human CD45+ cells (A, B), or human CD45-gated cells that were stained with biotinylated anti-Fcγ followed by SA-PE to detect the CAR (B), are indicated in each histogram. C, Quantitative bioluminescence imaging of tumor growth in Mouse 1 (top) and Mouse 2 (bottom) over time. Mean flux levels of luciferase activity were measured. Dashed lines represent day of T cell treatment. (PDF) [file pone.0146885.s001.pdf]
